# The first case report of rabies in a grey wolf (*Canis lupus*) in Chaharmahal and Bakhtiari Province (Iran)

**DOI:** 10.1002/vms3.755

**Published:** 2022-02-03

**Authors:** Mohammadreza Ghorani, Fahime Eslami, Ghafar Jafari

**Affiliations:** ^1^ Faculty of Veterinary Medicine Pathobiology Department University of Tabriz Tabriz Iran; ^2^ Chaharmahal and Bakhtiari Provincial Office of Department of Environment Shahrekord Iran

**Keywords:** FAT, rabies, wildlife, wolf

## Abstract

Rabies is an acute fatal viral encephalitis usually transmitted from animals to men following domestic and wild animal bites. Rabies is endemic in Iran. It is the most important zoonotic disease in the country. Here, we describe a case report of grey wolf rabies in Iran. One grey wolf in Chaharmahal and Bakhtiari province showed signs of rabies. Clinical signs were characterised by increased sensitivity, ferocity, restlessness, and depression is accompanied by lethargy. After a while, the animal died. The brain samples were taken from the wolf soon after death. The sample was refrigerated and transported fresh on ice to the laboratory. Fluorescent antibody technique (FAT) confirmed rabies infection in the wolf. Prevention and control of this fatal disease require a sensitive surveillance system to follow suspected animal and human rabies cases thoroughly through the improved reporting system, which contains the history of exposure, clinical examinations, symptoms, and laboratory results. Rapid and accurate diagnosis of rabies is very important due to its zoonotic and public health.

## 1 INTRODUCTION

Rabies is a fatal disease for warm‐blooded vertebrates, which causes central nervous system infection, paralysis, and death (Niezgoda, 2002) and is caused by a group of neurotropic viruses *Rhabdoviridae*, *Lyssavirus* genus. It has only one antigenic type and is transmitted from animals to humans by the bite of the affected animals (Radostits, 2007). Based on studies undertaken during the past few decades, there is evidence that the main reservoir for rabies is wolves. The incidence of rabies in humans and animals is increasing every year (Janani et al., 2008). Sylvatic and urban forms are the two features of the disease that appear in extreme and mild degrees of ferocity, respectively (Radostits, 2007). Although rabies in dromedaries has supposedly been observed in many African and Asian countries, little has been published on this subject (Esmaeili et al., 2012). On average, nine people die of rabies in Iran every year (Simani et al., 2004). However, the number of rabies cases and deaths seems to be greatly underestimated (Leylabadlo & Baghi, 2020). Sylvatic and urban forms are two different features of rabies and are mainly transmitted by wolves and dogs. Most positive cases have been due to dogs and ruminants (Simani, 2003). Rabies is endemic in the wildlife in Iran, where infection of domestic livestock is frequent (Esfandiari et al., 2010). According to the above, management of this problem is one of the most important priorities of Iran's Health Ministry.

The present paper described a case report of rabies in a grey wolf.

## 2 MATERIALS AND METHODS

### 2.1 History

**FIGURE 1:**
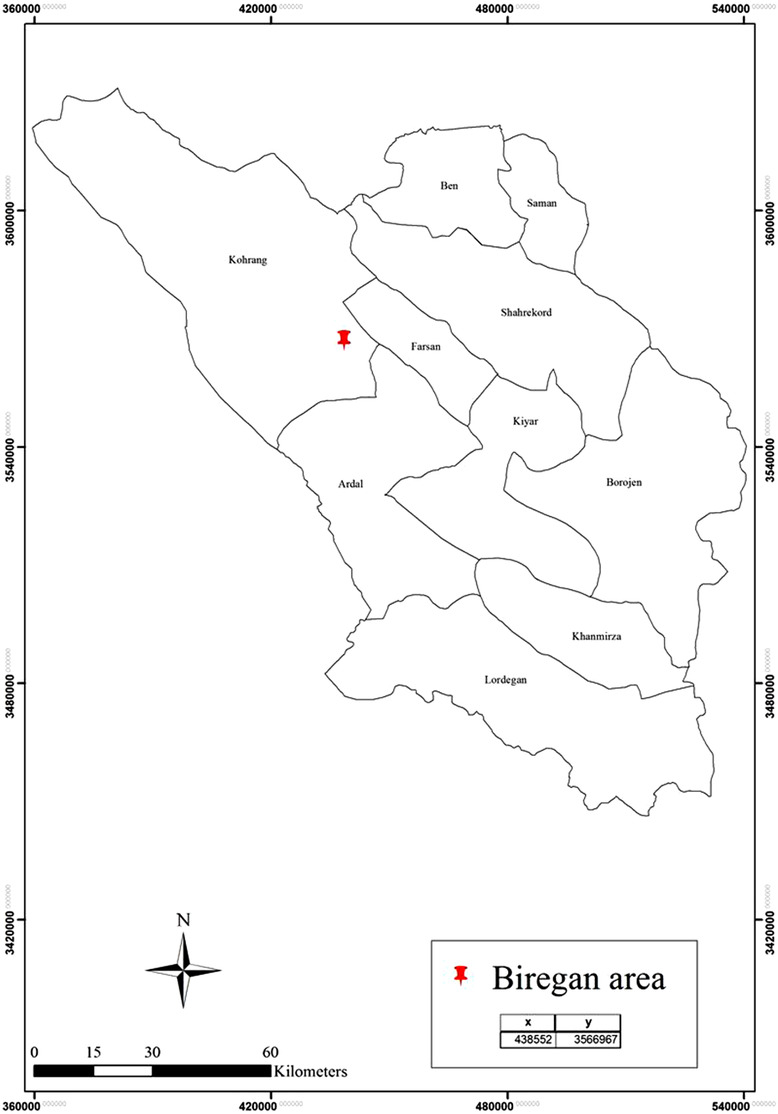
The geographical location of rabid grey wolf

In May 2021, in the Biregan area (Chaharmahal and Bakhtiari province; Figure 1), a grey wolf that could not escape was seen by Chaharmahal and Bakhtiari Provincial Office of Department of Environment. The wolf had been seen near the village, but there were no reports of attacks on humans or other animals. The wolf was sent to the Iran Veterinary Organization, but after a while, the animal died. To determine the cause of death, we took a rabies test, which revealed that the animal had rabies.

### 2.2 Physical features

The male wolf was about 7 years old according to the patterns of tooth wear (Gipson et al., 2000). The head and body length of the wolf is 98 cm, tail length is 37 cm, body height is 73 cm and body weight is 54 kg.

### 2.3 Laboratory investigations

The brain samples were taken from the wolf soon after death. The sample was refrigerated and transported fresh on ice to the laboratory. Impressions of tissue samples from the brain stem, hypothalamus, cerebellum and the hippocampus (Ammon's horns) were examined for rabies infection using FAT. FAT examination was applied on the fresh samples as well as described by WHO & OIE (Dean, 1996; OIE, 2008) with Iran Veterinary Organization.

## 3 RESULTS

**FIGURE 2:**
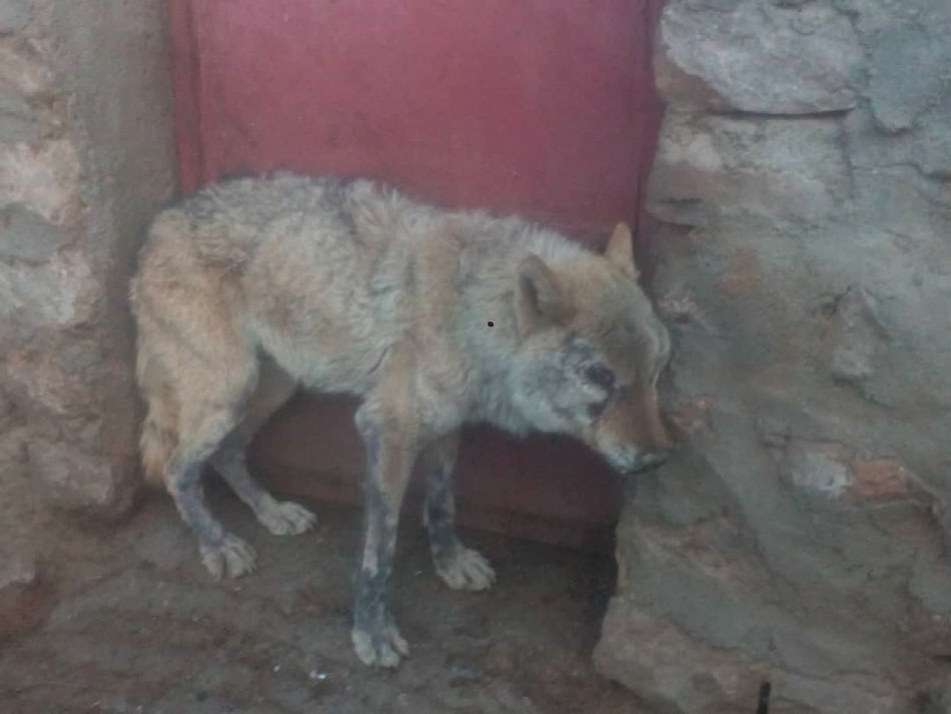
Rabid grey wolf

**FIGURE 3:**
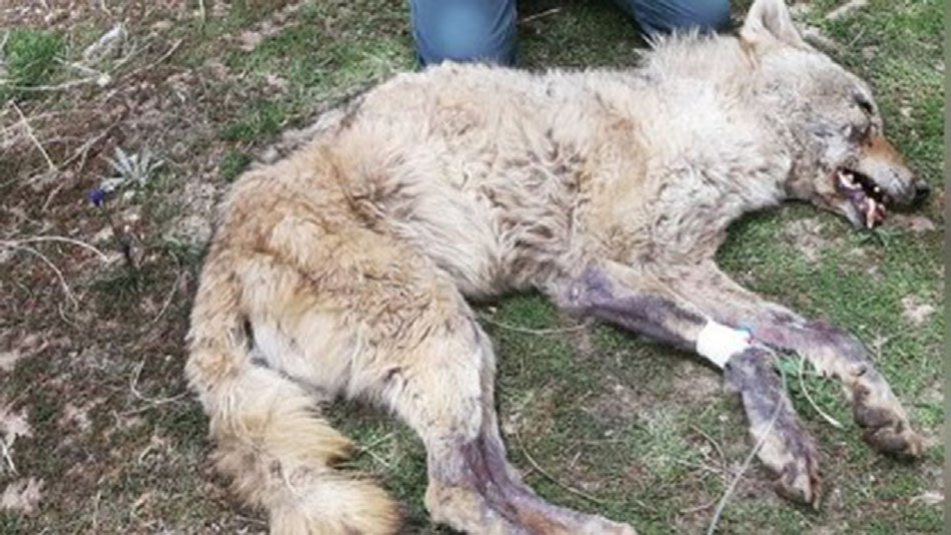
Death rabid wolf

**FIGURE 4:**
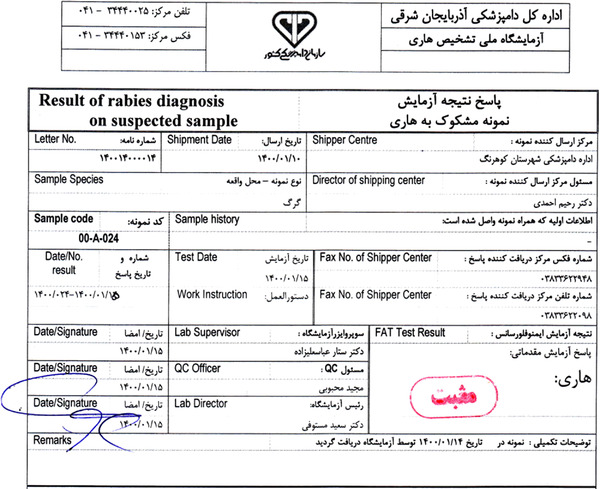
The result of rabid wolf

The grey wolf was lethargic and could not escape (Figure 2). The wolf was not afraid of humans and herding dogs. Depression showed with lethargy. After two hours, the wolf died. In a later stage, the rabid wolf became paralysed continuously till death (Figure 3). The samples were positive for rabies. The result of FAT is shown in Figure 4.

## 4 DISCUSSION

Rabies is an acute viral zoonotic disease that causes encephalitis in mammals (Lafon, 2005; Rupprecht et al., 2010). Wolves are extremely susceptible to rabies (Radostits OM, 2007). Studies of reservoirs of rabies in different parts of Iran exhibit that dog, fox and jackal are the most common reservoirs of the disease in northern areas, although wolves as the predominant ones in western and northwestern parts (Simani, 2003). The species of animals most likely to be infected with rabies vary from region to region. In the Caspian littoral, jackals and stray dogs are the most important rabid animals, while, in the mountainous areas of the central plateau, foxes and wolves play a more important role (Bokaei et al., 2009). In another report on animal rabies in Kerman province during 1993–2003, there was a significant seasonal variation in the number of suspected and confirmed rabid animals, and the peaks were in winter and autumn. The grey wolf is found in most habitats of Iran, from dense forests to desert areas. Although it is widely distributed in Iran, it is relatively rare. It is more abundant in the western and northwestern regions of Iran. The grey wolf is on the IUCN (International Union for Conservation of Nature) Red List of Low Concerned Species or LCs.

## 5 CONCLUSION

However, investigations performed showed that in many cases, wolves were responsible for rabies transmission to humans. Because of the potential feasibility of oral rabies vaccination, is proposed a pilot study to evaluate the use of an oral rabies vaccine for immunisation of wildlife to control rabies in Iran.

The importance of this case report is that, first, grey wolves are endangered animals and, second, because wolves live far away from humans, reports of rabies in wolves are rare and usually humans or domestic animals that were bite with rabid wolves have been reported.

Prevention and control of this fatal disease require a sensitive surveillance system to follow suspected animal and human rabies cases thoroughly through the improved reporting system, which contains the history of exposure, clinical examinations, symptoms and laboratory results. Rapid and accurate diagnosis of rabies is very important due to its zoonotic and public health.

## CONFLICT OF INTERESTS

The authors declare that there is no conflict of interest.

## ETHICAL APPROVAL

This study did not require an ethics license.

## AUTHOR CONTRIBUTIONS

Mohammadreza Ghorani: investigator and manuscript preparation; Fahime Eslami: sampling and data recording; Ghafar Jafari: sampling and data recording.

### PEER REVIEW

The peer review history for this article is available at https://publons.com/publon/10.1002/vms3.755.

## Data Availability

The author has provided the required data availability statement, and if applicable, included functional and accurate links to said data therein.
